# Long-term Dental Anomalies after Pediatric Cancer Treatment in Children

**DOI:** 10.4274/tjh.galenos.2018.2018.0248

**Published:** 2019-08-02

**Authors:** Gülser Kılınç, Gülçin Bulut, Fahinur Ertuğrul, Hale Ören, Bengü Demirağ, Ayşe Demiral, Serap Aksoylar, Emine Serra Kamer, Hülya Ellidokuz, Nur Olgun

**Affiliations:** 1 Dokuz Eylül University Faculty of Medicine, Department of Pediatric Dentistry, İzmir, Turkey; 2 İzmir Training Dental Hospital, Clinic of Pediatric Dentistry, İzmir, Turkey; 3 Ege University Faculty of Dentistry, Department of Pedodontics, İzmir, Turkey; 4 Dokuz Eylül University Faculty of Medicine, Department of Pediatric Hematology, İzmir, Turkey; 5 Behçet Uz Children’s Hospital, Clinic of Pediatric Hematology and Oncology, İzmir, Turkey; 6 Dokuz Eylül University Faculty of Medicine, Department of Radiation Oncology, İzmir, Turkey; 7 Ege University Faculty of Medicine, Department of Pediatric Hematology, İzmir, Turkey; 8 Ege University Faculty of Medicine, Department of Radiation Oncology, İzmir, Turkey; 9 Dokuz Eylül University, Institute of Oncology, Department of Preventive Oncology, İzmir, Turkey; 10 Dokuz Eylül University, Institute of Oncology, Department of Pediatric Oncology, İzmir, Turkey

**Keywords:** Cancer, Children, Dental anomalies, Hypodontia, Microdontia, Root malformation, Enamel defect

## Abstract

**Objective:**

The aim of this study is to determine the frequency of dental anomalies (DAs) (microdontia, hypodontia, hyperdontia, enamel defect, root malformation) in pediatric cancer patients at the ages <5 years and between 5 and 7 years, and understand their relationship with the received therapy.

**Materials and Methods:**

Pediatric patients who were diagnosed with cancer and treated before the age of 7 years were investigated in a case- control design. The study included 93 pediatric patients whose ages at diagnosis were between 9 months and 7 years and whose treatments were completed before 5-8 years. Group A consisted of patients in the age range of 9 months to 4 years and Group B consisted of patients in the age range of 5-7 years. Seventy-two siblings with compatible dental age ranges were included in the control group. For both groups, intraoral examinations were performed and panoramic radiographs were taken.

**Results:**

Among the 93 pediatric patients, the mean age was 9.54±1.25 (range: 8-13 years) and 48 (51.6%) patients were male. The most common diagnosis was hematologic malignancy with a rate of 65.5%. At least one DA was detected in 7 (9.7%) individuals of the control group and in 78 (83.9%) of the patient group. While the patients in the study group had all kinds of DAs, those in the control group had only enamel defects. The rates of microdontia (p=0.077) and hypodontia (p=0.058) were detected to be significantly higher in Group A than in Group B. Root malformation was more common in patients receiving chemotherapy and radiotherapy than in those receiving only chemotherapy (p=0.006).

**Conclusion:**

In this study it was found that the pediatric patients who received cancer treatment before the age of 7 years constituted a high-risk group for DAs. The frequencies of microdontia and hypodontia were increased even more when the patient was treated for cancer before 5 years of age.

## Abstract (tr)

**Amaç:**

Çalışmanın amacı <5 ve 5-7 yaş arası kanser tanısı alıp tedavi görmüş hastalarda diş anomalileri (DA) (mikrodonti, hipodonti, hiperdonti, mine kusuru, kök şekil bozukluğu) sıklığını belirlemek ve alınan tedavi ile ilişkini saptamaktır.

**Gereç ve Yöntemler:**

Kanser tanısı alıp 7 yaş öncesi tedavi görmüş çocuk hastalar olgu kontrol yöntemiyle araştırıldı. Kanser tedavisinin üzerinden en az 5-8 yıl geçmiş, tanı yaşı 9 ay ile 7 yıl arasında değişen, 93 hasta çalışmaya dahil edildi. Grup A 9 ay-4 yaş arasındaki hastalardan, Grup B 5-7 yaş arasındaki hastalardan oluşuyordu. Kontrol grubu olarak hastaların yaş aralığı uygun 72 kardeşi alındı. Hasta ve kontrol grubunun ağız içi muayeneleri yapıldı ve panoramik radyografileri alındı.

**Bulgular:**

Doksan üç hastanın yaş ortalaması 9,54±1,25 (dağılım 8-13 yıl) ve 48’i (%51,6) erkekti. En sık rastlanan tanı, %65,5 oranında hematolojik malignitelerdir. En az bir tane DA, hasta grubunun 78’inde (%83,9) ve kontrol grubunun 7’sinde (%9,7) saptandı. Çalışma grubundaki hastalarda her çeşit DA görülürken, kontrol grubunda sadece mine kusuru vardı. Grup A’da mikrodonti (p=0,077), hipodonti (p=0,058) oranlarının, Grup B’ye göre daha yüksek olduğu saptandı. Kök şekil bozukluğu kemoterapi ve radyoterapi alan hastalarda sadece kemoterapi alanlara göre daha fazla görüldü (p=0,006).

**Sonuç:**

Bu çalışmada 7 yaşından önce kanser tedavisi gören hastaların DA’lar yönünden yüksek riskli grup oluşturduğu saptanmıştır. Hastalar 5 yaşından önce kanser tedavisi gördüğünde mikrodontia ve hipodontinin sıklığı daha da artmıştır.

## Introduction

Malignant tumors are the second most common cause of death in children around the world [1,2,3]. Various late side effects can develop in these patients after cancer treatment. Late side effects are defined as permanent changes caused by disease, treatment, or both [1,2,3,4,5]. It has been reported that at least one side effect and related health problems are observed in approximately 40% of children receiving cancer treatment [6,7,8,9,10,11]. Most of these late side effects are not very serious, but they can still cause functional and aesthetic problems later in life, which may cause a decrease in quality of life [7].

The most common types of pediatric cancers are leukemia, central nervous system tumors, and lymphomas [1]. Chemotherapy (CT) and/or radiotherapy (RT) are usually the treatment of choice in these diseases. Most anti-cancer drugs used for cancer treatment block the growth of cancer cells owing to their cytostatic and cytotoxic effects and also enable these cells to be destroyed [1,2]. Previous animal studies have shown dental development disturbances induced by vincristine, vinblastine, doxorubicin, and cyclophosphamide [12,13]. RT can also cause disturbances in dental development in children; however, the minimal RT dose necessary to cause changes in dental development is unknown. On the other hand, researchers reported that a dose of 10 Gy RT will cause permanent changes in mature ameloblasts and a dose of 30 Gy is enough to stop dental development [13,14]. Therefore, the risk of dental anomalies (DAs) as a long-term side effect is quite high in children after cancer treatment [3,5,6,7]. The frequency and severity of DAs can vary depending on age at diagnosis, type and dose of chemotherapeutic agent used, RT total and fraction dose, and volume of oral cavity involved in the RT field [3,15,16,17,18].

It is known that DAs are more common among children who have received cancer treatment at an earlier age, which is usually before the age of 5 years [3,6,7,8]. On the other hand, the ages of 4-5 years are considered critical for tooth development [7]. For this reason, it is emphasized that it is very important to investigate possible DAs in pediatric patients who have received cancer treatment before the age of 5 years [6,7].

There are limited data on the long-term effects of cancer therapy on dental growth in pediatric patients in Turkey. The aim of this study is to determine the frequency of DAs in pediatric patients who were treated for cancer and to compare these patients with their siblings with regard to the frequency of DAs.

## Materials and Methods

### Study Population

### Patients

Pediatric patients who were diagnosed with cancer and treated in the Departments of Pediatric Hematology and Oncology and Radiation Oncology at Dokuz Eylül University and Ege University and in the Outpatient Clinic of Hematology and Oncology at Behçet Uz Children’s Hospital between January 2000 and December 2010 were included in the study. The first signs of root development in permanent teeth are generally observed on panoramic radiographs beginning approximately at the age of 3 to 7.5 years [6,9]. For that reason, patients with an age over 8 years were included in the study and the dental examinations were made between 5 and 8 years after cancer therapy. The patient population was divided into two groups according to the critical age for dental growth and previously published data: Group A consisted of patients in the age range of 9 months to 4 years and Group B consisted of patients in the age range of 5-7 years [7,8]. Leukemia, lymphoma, and Langerhans cell histiocytosis were categorized as lymphoproliferative diseases (LTs) and the remaining cancers were classified as solid cancers (STs). Patients were treated according to appropriate international CT protocols depending on their cancer diagnoses [19].

### Controls

Among 85 siblings of the 93 treated patients, 72 healthy siblings (8 to 16 years) were included in the control group. This study was approved by the ethics review committee of Dokuz Eylül University, Faculty of Medicine, İzmir, Turkey. All participants and their parents were given verbal information about the study and written informed consent was obtained from the parents.

### Clinical and Radiographic Examination for the Diagnosis of DAs

Intraoral examinations of all patients and controls were performed in a dental clinic environment. All teeth and their surfaces were examined by one pediatric dentist (the first author). Panoramic radiographs (Castellini X-Pan 85 2D) were taken just after intraoral examination in the same session. The panoramic radiographs were analyzed to determine the number of present permanent teeth and the changes in the size of the tooth crown or the root structure.

Teeth with short roots or V-shaped roots were evaluated as root malformations (RMs). Hölttä’s Defect Index was used for the assessment of root length as previously described [9,17]. Teeth for which the ratio between the root and crown length was below 1.6 were evaluated as short-rooted teeth. If a tooth was half the size of other teeth in the same group, the condition was accepted as microdontia [1,7,17]. The absence of a tooth or tooth germ in intraoral examination and in the panoramic radiograph without a history of extraction was evaluated as hypodontia. While assessing hypodontia, classification was performed as the absence of a single tooth, absence of 2-5 teeth, and absence of 6 and more teeth (oligodontia) [7]. The presence of white/cream and colored opacities or hypoplasia on the enamel of >2 mm was considered as enamel defect (ED). The condition of having supernumerary teeth in the dental arch was classified as hyperdontia.

Dental findings of all patients and controls in the study were shared with their families. All patients were later followed for treatment of the DAs in our clinics.

### Statistical Analysis

SPSS 15.0 for Windows (SPSS Inc., Chicago, IL, USA) was used in statistical analyses. Differences in clinical variables were evaluated using the chi-square test or Fisher exact test for qualitative variables. A value of p<0.05 was considered statistically significant.

## Results

A total of 93 children treated for cancer were included in the study. The mean age was 9.54±1.25 (range: 8-13) years and 48 (51.6%) patients were male. The mean age at cancer treatment was 3.75±2.01 years (range: 9 months to 7 years). The distribution of cancer types is presented in Table 1. The mean age of the control group was 10.60±2.40 (range: 8-16) years. No statistically significant difference was detected in patients and control groups in terms of their age and sex (p>0.05).

Seventy-eight children (83.9%) had at least one DA. ED was detected in 90 teeth of 22 patients (23.7%) in the treatment group and in 20 teeth of 7 patients (9.7%) in the control group (p=0.009).

Group A consisted of 59 patients and Group B consisted of 34 patients. The total number of DAs was not different between the groups; however the rates of microdontia and hypodontia were higher in Group A than in Group B (p=0.077 and p=0.058, respectively) (Table 2). The rates of RM and ED were similar in both groups.

No statistically significant difference was detected in patients in terms of sex and frequency of DAs (microdontia, hypodontia, hyperdontia, ED, and RM) (p>0.05) (Table 3).

The frequency of DAs was 80.6% in the ST, 85.5% in the LT, 81.0% in the CT, and 88.6% in the CT+RT groups. There was no significant difference in terms of tumor type and method of treatment (p=0.790 and p=0.338, respectively). However, RMs were observed to be more common in patients receiving CT+RT than in those receiving only CT (p=0.006). When patients receiving CT+RT to the head-neck region were analyzed (n=24), the rate of RMs significantly increased (p=0.001). According to the dose of RT (<20 Gy vs. ≥20 Gy) in 24 patients receiving RT to the head-neck region, only the rate of RMs was observed to significantly increase in parallel with dose (p=0.013) (Table 4).

In total, 413 teeth of the patients and 20 teeth of the controls were affected by DAs. The number of teeth with DAs was quite high in the patients undergoing cancer treatment (Table 5). While the numbers of teeth with microdontia and hypodontia were higher in Group A, the numbers of teeth with ED and RMs were close in Groups A and B.

Microdontia was more frequently found among second incisors, first and second premolars, and second molars in Group A compared to Group B. Hypodontia was often found among second incisors, first and second premolars, and second molars in Group A. RMs were almost equally distributed among all classes of teeth in Groups A and B, but they were more often found in the lateral or central incisors and first molars in Group A while in the first and second premolars in Group B. The ED rate was detected to be similar in these groups. Hyperdontia was observed in the right-left mandibular first and second premolars of only one patient in Group A.

## Discussion

Patients receiving cancer treatment in childhood are prone to development of DAs [3,7,8,18,20]. In our study, the overall rate of DAs was found to be 83.9%. There is a wide variation of the rate of DAs in the literature. Despite the presence of studies reporting rates similar to ours (82.9%-89.1%) [3,6,21], some others reported much lower rates (29%-62.3%) [7,20]. These differences are attributed to treatment age, CT protocols that are applied, and the presence of RT to the head and neck [9,22].

In our study, the rates of occurrence of DAs in Group A and Group B were similar. This result can be explained by the fact that dental development between 1 and 7 years is very active. In studies conducted on rats and hamsters, it has been biochemically and histologically demonstrated that chemotherapeutic agents affect developing teeth much more than developed teeth [10,23,24,25].

Maguire et al. [26] reported the absence of a statistically significant difference in the rate of DAs between leukemia and ST cases. Similar results were also found in our study. We found no significant differences between the patients receiving CT and those receiving CT+RT in terms of DAs, except for RM. Maciel et al. [27] reported that, in patients with acute lymphoblastic leukemia, the mean number of teeth with DAs was higher in the group undergoing conventional CT+RT of the whole cranium than in the group undergoing only conventional CT, but the difference was not statistically significant. In our study, when the frequency of DA was analyzed considering the dose of RT (<20 Gy vs. ≥20 Gy) in patients receiving RT to the head-neck region, the rate of RM was significantly increased with dose. Similarly, Kaste et al. [15] reported a dose-dependent risk of having at least one DA among 9308 pediatric cancer survivors; exposure of the jaw to RT doses exceeding 20 Gy contributed to a 4- to 10-fold higher risk of developing DAs [15].

Several studies reported a prevalence of microdontia ranging from 7% to 78% in childhood cancer survivors [6,17,18,21,26,27,28,29]. In our study, the rate of occurrence of microdontia was 64.5%. The rate of microdontia was previously reported to be 0.5% in healthy Turkish children [30]. Hölttä et al. [17] found this rate as 75% in children younger than 3 years, 60% in those between the ages of 3 and 5 years, and 13% in those older than 5 years. Wilberg et al. [28] reported the rate of 54.0% in children with leukemia at the age of ≤5 years. Proc et al. [7] stated that microdontia was mostly observed in the first and second premolars in pediatric patients whose treatments were started at the age of ≤30 months. In our study, the rate of occurrence of microdontia was higher in Group A and first and second premolars were observed to be affected more frequently.

Anti-cancer treatment applied to patients causes hypodontia and its prevalence varies between 6% and 44% [8,9,20,31]. Pedersen et al. [1] reported a strong relationship between microdontia and hypodontia. In our study, the rate of occurrence of hypodontia was 22.6%. Nishimura et al. [18] found the rates of hypodontia and microdontia to be higher in children at the age of ≤4 years. In our study, the rates of microdontia and hypodontia were higher in children younger than 5 years old. While hypodontia is mostly observed in lateral incisors, second premolars, and third molars in healthy individuals [32,33], it is more frequent in second premolars and second molars in patients receiving cancer treatment [7]. The reason for hypodontia to occur in some tooth groups more commonly is that the time of calcification differs for different kinds of teeth [7,34].

In small children, application of CT and/or RT during odontogenesis can delay the development of Hertwig’s epithelial root sheath. For this reason, higher rates of RM are reported for these patients [3,22]. Researchers have found the rate of occurrence of RM to be between 11.5% and 16.1% and it is more frequently encountered in patients older than 4 years [7,9,35]. RMs were significantly more common among first and second premolars and first and second molars. The development of tooth roots begins approximately at the ages of 3 and 4 years and finishes at the age of 16 years [36]. Although the rate of RM was higher in Group B (32.4%) than in Group A (22.0%), there was no statistically significant difference. Similarly, Maciel et al. [27] found no significant difference between patients older than 5 years and patients ≤5 years old in terms of RMs. In our study, RMs were mostly in the central, lateral, and first molars in Group A and in the first and second premolars in Group B. Our findings are consistent with the developmental periods of tooth roots [36].

ED is the most common defect in the general population. It is the result of ameloblastic damage as far as it concerns the reproductive and secretory function, the membrane permeability, and calcium exchange across the membrane. Studies have reported that children in long-term remission of a malignant disease display a high incidence of ED [3,8]. It is mentioned that chemotherapeutic agents such as vincristine, vinblastine, and cyclophosphamide affect odontogenesis much more [8,10]. In our study, the rate of ED was higher in the patients (23.7%) than in their siblings (9.7%) and the difference was statistically significant. Similarly, the frequency of ED was higher in patients undergoing cancer treatment compared to their siblings in some other studies [26,31,37].

Hyperdontia was encountered in two teeth of a patient receiving treatment for retinoblastoma. Maciel et al. [27] stated that they found hyperdontia in their study, but its incidence was quite low and there was no difference between the study group and control group.

### Study Limitations

Our study has several limitations. First, the number of patients is relatively small and our results cannot be generalized to all pediatric cancer patients. However, we have included patients from 3 centers. Second, we did not have detailed information about patients’ routine oral and dental care, which might have an impact on DAs. The strength of the present study is the time period between cancer therapy and dental evaluation. We think that this duration is enough for confirmation of the development of DAs after cancer therapy, especially in patients aged less than 5 years. Another important point is that we have included patients’ siblings as a control group in the study in order to compare possible genetic effects.

## Conclusion

Pediatric patients undergoing cancer treatment at early ages constitute a high-risk group in terms of dental complications. Therefore, parents of pediatric patients undergoing cancer treatment should be informed by pediatric oncologists-hematologists and radiation oncologists about dental abnormalities that can develop in the future. Moreover, pediatric dentists should be integral members in the management of all children receiving cancer therapy. We think that periodic dental control and protective measures, at least twice a year, are essential and should be performed both during and after cancer therapy for pediatric patients.

## Figures and Tables

**Table 1:**
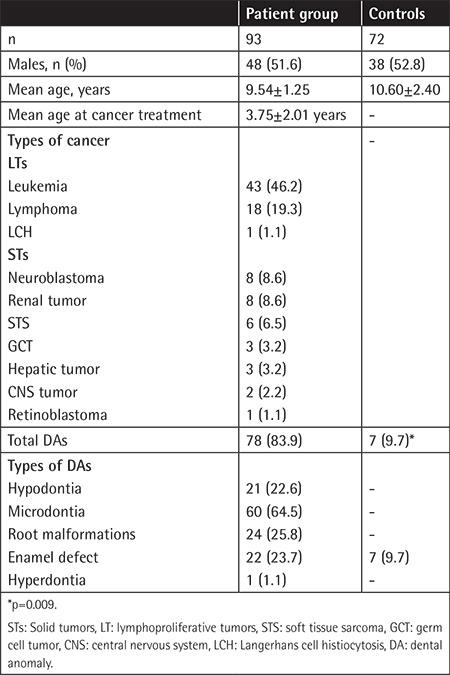
Patient and control group characteristics.

**Table 2:**
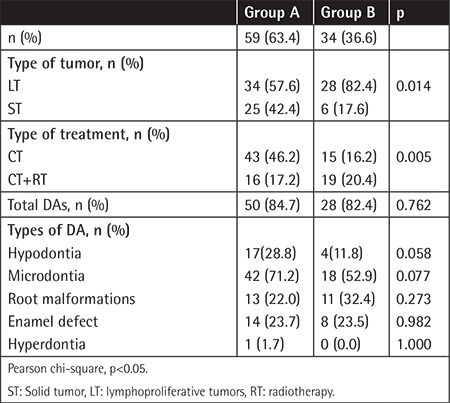
Comparison of patient groups according to age at treatment.

**Table 3:**
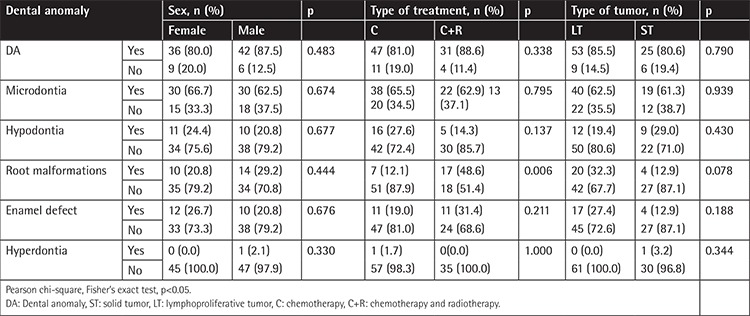
Comparison of the frequencies of dental anomalies according to sex, type of treatment, and tumor type.

**Table 4:**
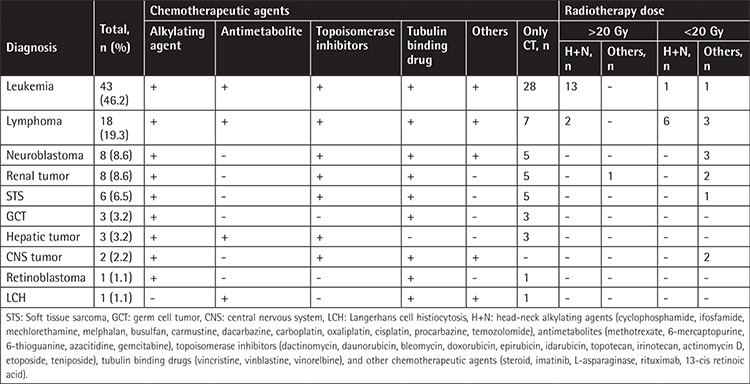
Treatment characteristics.

**Table 5:**
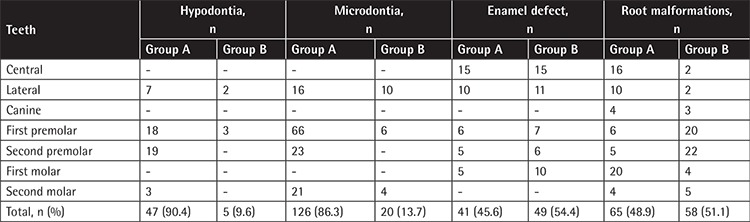
Distribution of teeth with anomalies.
